# Food insecurity and sleep deficiency among older Filipinos: the mediating roles of depressive symptoms and frailty phenotypes

**DOI:** 10.7189/jogh.15.04235

**Published:** 2025-08-29

**Authors:** Divine Grace C Domingo, Tuo-Yu Chen, Grace Cruz, Philile S Mgabhi, Kian Lee Lim, Chrispin Mandiwa, Yasuhiko Saito

**Affiliations:** 1 Department of Global Health and Health Security, Taipei Medical University, Taipei, Taiwan; 2 Institute of Human Nutrition and Food, University of the Philippines Los Baños, Laguna, Philippines; 3 Institute of European and American Studies, Academia Sinica, Taipei, Taiwan; 4 Population Institute, University of the Philippines Diliman, Quezon City, Philippines; 5 College of Economics, Nihon University, Tokyo, Japan; 6 Economic Research Institute for ASEAN and East Asia, Jakarta, Indonesia

## Abstract

**Background:**

Although research has shown that food insecurity may lead to sleep problems due to poor mental health, it remains unclear whether physical health mediates this relationship. We investigated whether depressive symptoms and frailty phenotypes mediated the association of food insecurity with sleep deficiency among community-dwelling older adults in the Philippines.

**Methods:**

We analysed data from the baseline survey of a longitudinal study of ageing and health in the Philippines involving a nationally representative sample of older adults aged ≥60 years (n = 3599). Sleep deficiency was conceptualised as self-reported sleeping of <6 hours, complaining about falling and staying asleep, and/or having non-restorative sleep. Food insecurity was defined as household hunger and lack of food over the past three months. Frailty phenotypes were operationalised using modified Fried’s criteria, and depressive symptoms using the Centre for Epidemiological Studies-Depression Scale. Covariates included sociodemographics (age, sex, education, wealth, living arrangements, and urbanity), health (pain, chronic diseases, and body mass index), and lifestyles (naps, smoking, and drinking). Mediation analysis using the PROCESS macro was conducted to estimate the direct and indirect effects in the moderated mediation models.

**Results:**

Bootstrapping results showed significant indirect effects of food insecurity on sleep deficiency through depressive symptoms (bootstrap estimate (b) = 0.09, standard error (SE) = 0.02; 95% confidence interval (CI) = 0.05, 0.14) and frailty phenotypes (b = 0.03, SE = 0.01; 95% CI = 0.01, 0.06). However, the direct effect of food insecurity on sleep deficiency was insignificant (b = 0.31, SE = 0.16; 95% CI = –0.003, 0.64).

**Conclusions:**

The results indicated that food insecurity is associated with sleep deficiency, with frailty phenotype and depressive symptoms playing a potential mediating role. However, further research is needed to establish causal pathways. Preventing frailty and depression may help improve sleep health among individuals who are food insecure.

Sleep problems broadly include issues with the quality, timing, and amount of sleep [1], with one common problem being sleep deficiency [2–6], which refers to an insufficient amount or poor quality of sleep compared to what is necessary for optimal health, cognitive function, and overall well-being [5,7]. However, sleep deficiency extends beyond inadequate sleep duration; it also encompasses sleepiness at inappropriate times, poor sleep quality, and sleep disorders that disrupt restorative sleep [6], and has been linked to poor physical and mental health and increased mortality rate [4–6]. Moreover, if left untreated, it can increase healthcare burdens [6,8]; *e.g.* Australia’s estimated cost of sleep disorders was around USD 10 billion in 2019–20 [8]. Hence, it is important to understand sleep problems and their associated factors among older adults to develop preventive strategies to enhance public health [9].

Food insecurity, a neglected public health issue, may affect sleep among older adults. It is considered a social determinant of health, defined as an economic and social condition at the household level characterised by restricted or uncertain access to sufficient nutritious food [10], which can lead to the limited ability of the body to support growth, development, and a healthy life [9]. Approximately 30% of the global population experienced moderate or severe food insecurity in 2023 [11]. Evidence also shows that the prevalence of food insecurity among older adults is on the rise [12]. Food insecurity may affect sleep because insufficient food intake can result in adverse health outcomes among adults, such as cardiometabolic risk [13,14] and psychological distress [15,16]. Previous studies have also demonstrated an association between food insecurity and poor sleep quantity and quality. For instance, one study found that adults in Mexico with severe household food insecurity were less likely to reach the recommended amount of sleep and experienced more days of sleep difficulties compared to food-secure households [17]. In another study, food insecurity was significantly associated with self-reported short sleep duration and poor sleep quality, although racial or ethnic differences were observed [18]. Still, in another study, food insecurity was significantly associated with objectively measured sleep duration, efficiency, and self-reported sleep quality [16]. Although a recent meta-analysis concluded that food insecurity was related to sleep problems among the adult population, the researchers found high heterogeneity across the included studies. They called for more research to understand the relationship between food insecurity and sleep problems and their underlying mechanisms [19].

Lee and colleagues [20] proposed a framework to explain why food insecurity might lead to sleep problems. In this framework, food insecurity can cause sleep problems via a psychological mechanism, such as depression, and a biobehavioural mechanism, such as poor nutritional status. Sociodemographic status (*e.g.* education) can affect these two pathways because they predetermine the quality and quantity of food consumed [21]. Two studies investigated the psychological mechanism [15,16]. In one study, psychological distress (*i.e.* depressive and anxiety symptoms and diabetes-related distress) significantly mediated the relationship between household food insecurity and sleep quality among Latinos with type 2 diabetes [15]. A similar finding was observed in another study, in which the researchers found that psychological distress partially mediated the effects of food insecurity on sleep quality among adults living in low-income neighbourhoods in Pennsylvania, USA. [16]. The biobehavioural mechanism has not been tested, although research suggests that such a relationship might exist. For instance, studies have shown that food insecurity is significantly related to poor nutritional status [22,23], which in turn has been linked to sleep problems [3,24].

Using Lee and colleagues’ framework [20], the current study investigated the psychological and biobehavioural mechanisms between food insecurity and sleep problems among community-dwelling older adults in the Philippines. Approximately 51 million Filipinos experienced food insecurity between 2020–22, the highest number in Southeast Asia [11]. A recent estimate also showed that one in ten Filipinos experienced food insecurity in 2022 [25], and according to the 2018 Longitudinal Study of Ageing and Health in the Philippines (LSAHP) wave one report, 13% of older Filipino households reported experiencing hunger in the past three months [26]. Filipino older adults are more vulnerable to food insecurity, particularly those whose livelihoods depend on agriculture [27]. Within food-insecure households, limited resources are often allocated preferentially to meet the nutritional needs of children, resulting in a lesser portion of food being allocated to older household members during meals [28]. Given that the older adult population is increasing in the Philippines [29], but research aiming to understand food insecurity and sleep problems in this population is scarce [30], examining the relationship between food insecurity and sleep problems among older Filipinos could expand the literature and provide unique insights for stakeholders to develop strategic plans to address the impacts of food insecurity.

Based on previous studies [15,16], depressive symptoms were proposed as mediators in the psychological pathway between food insecurity and sleep problems (**Figure 1**). It was hypothesised that older individuals who experienced food insecurity might have poorer sleep quality due to experiencing a higher number of depressive symptoms. Regarding the biobehavioural pathway, it has been proposed that food insecurity might lead to sleep problems via pronounced frailty phenotypes (*i.e.* weight loss, exhaustion, low physical activity, slowness, and weakness) [31]. Poor nutritional status positively correlates with frailty [32]. Research has also shown that food insecurity can lead to frailty among community-dwelling older adults [22,23,33]. In addition, frailty has been associated with increased sleep problems in this population [34,35]. Therefore, this study hypothesised that older individuals who experience food insecurity might experience sleep problems due to the presence of more frailty phenotypes.

**Figure 1:**
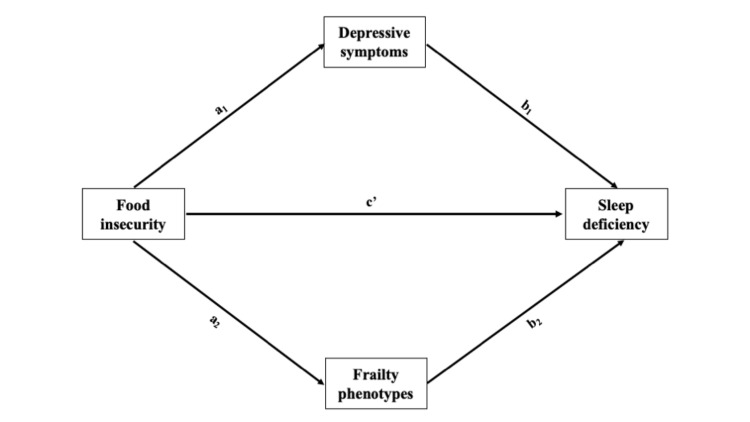
Conceptual model for the relationship between food insecurity and sleep deficiency via depressive symptoms and frailty phenotypes. According to this model, individuals who experience food insecurity may feel more depressed (a1), which could lead to sleep deficiency (b1). In a similar vein, individuals who experience food insecurity may experience more frailty phenotypes (a2), which could also cause sleep deficiency (b2). The total effects of food insecurity on sleep deficiency can be parcelled out as the indirect effects of food insecurity on sleep deficiency through depressive symptoms (a1 × b1), the indirect effects of food insecurity on sleep deficiency through frailty phenotypes (a2 × b2), and the remaining direct effects (c’).

## METHODS

### Data and respondents

We analysed the baseline survey data from the LSAHP, a study with a nationally representative sample of older Filipinos [26,36]. Details of the sampling design have been reported by Barrios and colleagues [37]. The 2018 LSAHP used a multistage sampling design to ensure representative coverage of older adults nationwide. In the first stage, provinces were stratified based on the 2015 census data, with provinces grouped into low, medium, and high older adult population categories. Systematic sampling was used to select provinces with proportional representation across regions (National Capital Region, Balance Luzon, Visayas, and Mindanao). In the second stage, *barangays* (villages) were selected using a probability proportional to the size of older adults. The target sample size was 6335 older adults, and 5985 individuals completed the interview (94% response rate).

Of the 5985 respondents, data from proxy respondents were excluded, as well as those with missing key variables. The final sample size was 3599. Compared to those who were excluded (n = 2386), the current sample was significantly younger (mean (x̄) age = 71.7 *vs.* x̄ = 75.0; (t-statistics (t) (n = 4553.98) = 14.86; *P* < 0.001) and had fewer females (62.8% *vs.* 65.5%; (Chi square (χ2) (1, N = 5989) = 4.6); *P* = 0.032), but education level was similar.

### Measures

#### Sleep deficiency

Because problems with sleep quantity and quality are critical, following previous studies [4,38], sleep deficiency, short sleep duration, and/or sleep insufficiency were used to conceptualise sleep problems. A short sleep duration was defined as sleeping six hours or less. Sleep insufficiency was assessed by sleep complaints and non-restorative sleep. Sleep complaints were assessed by asking respondents to rate their experiences with trouble falling asleep, waking up during the night, and being unable to sleep again after waking up too early in the morning on a four-point scale (most of the time, sometimes, rarely, and never). Individuals who responded most of the time or sometimes to any of the three sleep problems were considered to have sleep complaints. Non-restorative sleep was evaluated by asking the respondents the frequency (most of the time, sometimes, rarely, or never) of feeling rested in the morning. Responses of rarely or never were considered non-restorative sleep. We created a dummy variable to assess sleep deficiency, with one indicating individuals experiencing short sleep duration, sleep complaints, and/or non-restorative sleep.

#### Food insecurity

During the LSAHP interview, the respondents were asked, ‘In the last three months, did it happen even once that your household experienced hunger and did not have anything to eat?’ Individuals who answered yes were considered to be experiencing food insecurity.

#### Mediators

Frailty phenotypes and depressive symptoms were the mediators in this study. Frailty phenotypes were conceptualised using the modified Fried criteria used in previous studies, which is a widely accepted and validated frailty phenotype model characterised by weight loss, exhaustion, low physical activity, slowness, and weakness [2,39]. Weight loss was assessed using the ‘poor appetite’ item from the Centre for Epidemiological Studies Depression (CESD) scale. Exhaustion was defined using the items ‘everything is an effort’ or ‘could not get going’ from the CESD. Low physical activity was evaluated based on the respondents’ participation in walking, callisthenics, ballroom dancing, or gardening. Slowness was assessed by the respondents’ ability to walk 200–300 m, and weakness was evaluated by their ability to lift or carry five kg of groceries (Table S1 in the **Online Supplementary Document**). The total possible number of phenotypes (from zero to five) was used to indicate the degree of frailty [40].

Depressive symptoms were assessed using the 11-item CESD [41]. The responses ranged from zero (rarely/not at all) to two (often). The four items were excluded from the calculation of the total score (‘restless sleep,’ ‘poor appetite,’ ‘everything is an effort,’ and ‘could not get going’) to avoid variance circularity with frailty phenotypes and sleep deficiency. The total score ranged from 0–14.

### Covariates

We included as covariates sociodemographic information (age, sex, education, wealth status, living alone, living in urban area) [20,30,38,42], health status (pain, chronic conditions, body mass index (BMI)) [2,30,38], and health behaviours (naps, smoking, drinking) [2,30,38] related to food insecurity, sleep deficiency, depressive symptoms, and frailty phenotypes.

Sociodemographic information included age in years, sex (female, male (male as a reference group)), education (elementary level or lower (lower as a reference group)), high school, college, or higher), living alone (yes/no), and living in an urban area (yes/no). Wealth scores were calculated by weighted principal component analysis based on the ownership of the number and types of consumer durables (*e.g.* television and cars) and house characteristics (*i.e.* construction materials, sources of drinking water, and toilet facilities). The wealth scores were then ranked into quintiles to obtain the wealth index, with one indicating the poorest quintile and five indicating the highest quintile.

For health status, pain was assessed by asking whether the respondents were often troubled with pain (yes/no). If yes, they were further asked to rate the severity of the pain they experienced (mild, moderate, and severe). A four-level categorical variable of pain severity was created based on these two questions: no pain (reference group), mild pain, moderate pain, and severe pain. Chronic conditions were assessed by asking the respondents whether a doctor had ever told them that they had the following conditions: heart disease (*e.g.* angina, and myocardial infarction), heart attack, cancer, cerebrovascular disease, high blood pressure, diabetes, lung disease (*e.g.* asthma and emphysema), renal disease, liver disease, and arthritis. The total number of diagnosed chronic conditions respondents was calculated (zero to ten), with a higher number indicating more chronic conditions. BMI (kg/m^2^) was categorised into underweight (<18.5 kg/m^2^), normal (18.5–24.9 kg/m^2^; reference group), pre-obese (25.0–29.9 kg/m^2^), and obese (≥30 kg/m^2^) [43].

Three health behaviours were included. Nap habits were assessed by asking the respondents whether they took naps (yes/no). Smoking was assessed by asking the respondents whether they currently smoked (yes/no). If not, they were further asked whether they used to smoke. These two items created a three-level categorial variable: never smoked (reference group), ever smoked, and current smokers. Similar questions for drinking were asked and used to create a variable of drinking behaviour (never drank {reference group}, ever drank, and current drinkers).

### Statistical analysis

We used descriptive analyses to examine the sample characteristics. Independent *t* tests were used to investigate the differences in continuous variables between individuals with and without sleep deficiency, and χ^2^ tests were used for categorical variables. Parametric and non-parametric correlational analyses were used to investigate the relationships among the variables.

We tested the hypothesis on a parallel mediator model using model four of Hayes’s ‘PROCESS’ macro, version 4.1 [44] on SPSS, version 26 (IBM SPSS Statistics for Windows, Armonk, New York, USA). The PROCESS macro is an advanced tool for mediation analysis due to its capacity to accommodate non-normally distributed data. Previous research has used this approach to test mediating effects [45,46]. The PROCESS macro employs bootstrapping, which repeatedly resamples smaller subsets from the original data set to create a tailored distribution for estimating the indirect effect and confidence interval [47,48]. This method helps minimise type one errors and enhances the accuracy and reliability of mediation estimates compared to conventional approaches [48,49]. According to Hayes [45], a binary outcome variable would be tested using the log-odds metric in the PROCESS macro, and the interpretation of the results would be similar to a continuous outcome variable.

The PROCESS macro estimated the mediating effects based on the relationship between food insecurity and depressive symptoms, the effects of food insecurity on frailty phenotypes, and the effects of food insecurity, depressive symptoms, and frailty phenotypes on sleep deficiency, adjusting for covariates (**Figure 1**). A total of 5000 bootstrap samples with a 95% confidence interval (CI) were implemented to test the significance of indirect effects. A significant indirect effect does not contain zero in the 95% CI [44].

## RESULTS

### Sample characteristics

A total of 3599 participants were analysed in the study, with 2908 (81%) experiencing sleep deficiency and 691 (19%) having no sleep deficiency (**Table 1**). Several significant differences were observed between participants with and without sleep deficiency. Older adults with sleep deficiency were more likely to sleep <6 hours (41%), experience insomnia symptoms (89%), and report non-restorative sleep (21%). Additionally, food insecurity was observed to be significantly higher among those with sleep deficiency (15% *vs.* 5%, *P* < 0.001).

**Table 1:** Sample characteristics and their relationships with sleep deficiency (sampling weight applied)*

Characteristics	All participants (n = 3599)	No sleep deficiency (n = 691)	Sleep deficiency (n = 2908)	*P*-value
Sleep <6 h	32		41	
Any insomnia symptoms	72		89	
Not restorative sleep	17		21	
Sleep deficiency	80		100	
Food insecure	13	5	15	<0.001†
Depressive symptoms, x̄ (SD)	2.75 (2.24)	1.89 (1.90)	2.97 (2.27)	<0.001†
Frailty phenotypes, x̄ (SD)	1.53 (1.10)	1.32 (1.00)	1.58 (1.12)	<0.001†
Age in years, x̄ (SD)	68.16 (6.80)	68.04 (6.42)	68.18 (6.89)	0.571
Female	60	51	62	<0.001†
Education				0.007‡
*Elementary or lower*	72	71	72	
*High school*	20	19	21	
*College or above*	8	11	7	
Live alone	13	9	14	<0.001†
Live in an urban area	46	45	46	0.461
Wealth index, x̄ (SD)	2.93 (1.37)	3.15 (1.40)	2.88 (1.36)	<0.001†
Pain				<0.001†
*No*	68	80	65	
*Mild*	11	9	11	
*Moderate*	19	10	21	
*Severe*	2	1	3	
Chronic conditions, x̄ (SD)	1.22 (1.18)	0.99 (1.15)	1.28 (1.18)	<0.001†
BMI				0.004‡
*Underweight*	12	13	12	
*Normal*	55	49	56	
*Pre-obese*	25	29	24	
*Obesity*	8	9	8	
Nap	81	87	80	<0.001†
Smoking				0.043
*Never smoke*	57	54	58	
*Ever smoked*	25	25	25	
*Current smoker*	18	21	17	
Drinking				0.013
*Never drink*	50	47	50	
*Ever drank*	21	20	21	
*Current drinker*	30	33	29	

BMI – body mass index, SD – standard deviation, x̄ – mean

*Presented as % unless specified otherwise.

†*P* < 0.001.

‡*P* < 0.01.

There were significant differences in the mental and physical health characteristics of the participants. Older adults with sleep deficiency were observed to have higher mean (x̄) depressive symptom scores (x̄ = 2.97, standard deviation (SD) = 2.27 *vs.* x̄ = 1.89, SD = 1.90, *P* < 0.001). They were also more likely to report pain (35% mild to severe *vs.* 20% mild to severe, in those without sleep deficiency, *P* < 0.001) and exhibited a higher x̄ number of chronic conditions (x̄ = 1.28, SD = 1.18 *vs.* x̄ = 0.99, SD = 1.15, *P* < 0.001).

In terms of sociodemographic characteristics, older adults with sleep deficiency were more likely to be women (62% *vs.* 51%, *P* < 0.001), living alone (14% *vs.* 9%, *P* < 0.001), and belong to a lower wealth index score (x̄ = 2.88, SD = 1.36 *vs.* x̄ = 3.15, SD = 1.40, *P* < 0.001). There were no significant differences in the x̄ age of the participants (x̄ = 68.16, SD = 6.80 years) or their educational level, with the majority (72.0%) having completed elementary school or a lower level of education.

Regarding lifestyle factors, both groups exhibited high rates of napping, with 80% of those with sleep deficiency and 87% of those without sleep deficiency reporting this behaviour (*P* < 0.001). Smoking behaviour showed a slight non-significant difference between the groups (*P* = 0.013). Regarding BMI classification, more than half (51%) of older adults without sleep deficiency were classified as outside the normal BMI range – 13% underweight, 29% pre-obese, and 9% obese – compared to 44% of those with sleep deficiency, with 12% underweight, 24% pre-obese, and 8% obese. However, the BMI differences between the groups were not statistically significant (*P* = 0.004). The relationships among all studied variables can be found in Table S2 in the **Online Supplementary Document**. In general, the correlation coefficients of sleep deficiency, food insecurity, depressive symptoms, and frailty phenotypes with other variables ranged from 0.0 to 0.3, suggesting a low to moderate correlation.

### The indirect effects of food insecurity on sleep deficiency through depressive symptoms and frailty phenotypes

**Figure 2:**
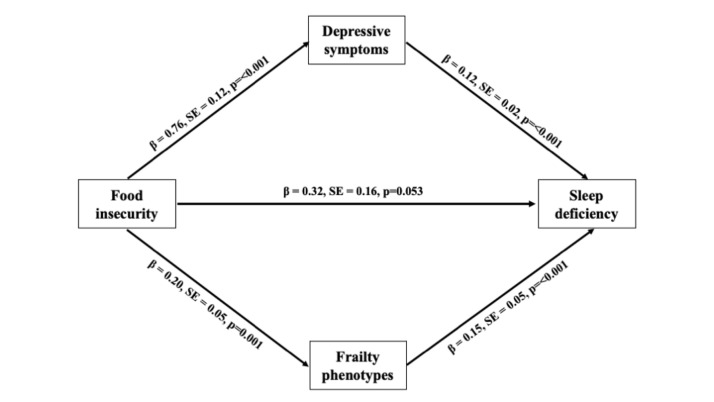
Mediational analysis examining the effects of food insecurity on sleep deficiency (binary outcome). The results from bootstrapping showed that the total indirect effect was 0.12 (SE = 0.03; 95% CI = 0.08, 0.18), the indirect effect through depressive symptoms was 0.09 (SE = 0.02; 95% CI = 0.01, 0.06), and the indirect effect through frailty phenotypes was 0.03 (SE = 0.03; 95% CI = 0.01, 0.06). These significant indirect effects suggested that food insecurity led to sleep deficiency because of the greater number of depressive symptoms and frailty phenotypes among community-dwelling older Filipinos. The model adjusted for age, sex, education, living arrangement, living in urban areas, wealth status, pain, chronic conditions, BMI, nap habits, smoking, and drinking. The model fit was significant, χ^2^ (22) = 194.57, *P* < 0.001, suggesting that there was a significant improvement in the fitted model compared to the null model. BMI – body mass index, CI – confidence interval, SE – standard error.

The mediational analysis examined whether food insecurity was indirectly associated with sleep deficiency through depressive symptoms and frailty phenotypes, adjusting for age, sex, education, living arrangement, wealth status, pain, chronic conditions, BMI, nap habit, smoking, and drinking. The first mediation pathway examined whether depressive symptoms mediate the relationship between food insecurity and sleep deficiency, testing the hypothesis that older adults experiencing food insecurity are more likely to experience higher depressive symptoms, which in turn contribute to sleep deficiency. The results indicated that food insecurity was significantly associated with a higher number of depressive symptoms (*β* = 0.76, *P* < 0.001), and depressive symptoms were significantly associated with a higher probability of having sleep deficiency (*β* = 0.12, *P* < 0.001) (**Figure 2**).

The second mediation pathway explored whether frailty phenotypes mediate the relationship between food insecurity and sleep deficiency, based on the hypothesis that older adults who are food insecure may experience pronounced frailty phenotypes, which subsequently increases their likelihood of experiencing sleep deficiency. The results demonstrated that food security was significantly associated with frailty phenotypes (*β* = 0.20, *P* < 0.001), and frailty phenotypes were significantly associated with a higher probability of having sleep deficiency (*β* = 0.15, *P* < 0.001).

Taken together, these findings support the notion that both depressive symptoms and frailty phenotypes play mediating roles in the relationship between food insecurity and sleep deficiency. However, the direct pathway from food insecurity to sleep deficiency was insignificant (*β* = 0.32, *P* = 0.05). The model adjusted for age, sex, education, living arrangement, residence in urban areas, wealth status, pain, chronic conditions, BMI, nap habits, smoking, and drinking. The model fit was significant, χ^2^ (22) = 194.57, *P* < 0.001, suggesting a substantial improvement in the fitted model compared to the null model (Table S3 in the **Online Supplementary Document****)**.

The results of the bootstrapping revealed that the total indirect effects from food insecurity to sleep deficiency through depressive symptoms and frailty phenotypes were significant (*β* = 0.12; 95% CI = 0.08, 0.18). The pathway from food security to sleep deficiency via depressive symptoms (*β* = 0.09; 95% CI = 0.05, 0.14) and frailty phenotypes (*β* = 0.03; 95% CI = 0.01, 0.06) were significant. The direct effect of food insecurity on sleep deficiency was not significant (**Table 2**).

**Table 2:** Indirect effects of food insecurity on sleep deficiency through depressive symptoms and frailty phenotypes*

Mediator	Effect of food insecurity on depressive symptoms (*β*)	SE	Effect of depressive symptoms on frailty phenotypes (*β*)	SE	Bootstrap estimate (*β*)	SE	95% CI
Depressive symptoms	0.76†	0.12	0.12†	0.02	0.09	0.02	0.05, 0.14
Frailty phenotypes	0.20‡	0.05	0.15†	0.05	0.03	0.01	0.01, 0.06
Total indirect effects	NA	NA	NA	NA	0.12	0.03	0.08, 0.18
Direct effect of food insecurity on sleep deficiency	NA	NA	NA	NA	0.31	0.16	–0.003, 0.64

CI – confidence interval, NA – not available, SE – standard error

*The model adjusted for age, sex, education, living arrangement, living in urban areas, wealth status, pain, chronic conditions, BMI, nap habits, smoking, and drinking.

†*P* < 0.001.

‡*P* < 0.01.

## DISCUSSION

In this study, we examined whether the association of food insecurity with sleep deficiency was mediated through depressive symptoms and frailty phenotypes among community-dwelling older Filipinos aged ≥60 years. A high prevalence of sleep deficiency (81%) was observed among Filipino older adults, which was much higher than previously reported estimates among community-dwelling older adults in Japan (26%) and Singapore (48%) [38]. Additionally, food insecurity was found to be significantly associated with a higher number of depressive symptoms and frailty phenotypes. Depressive symptoms and frailty phenotypes were significantly associated with sleep deficiency. Moreover, the results from bootstrapping showed that the indirect effects of food insecurity on sleep deficiency were significantly channelled through depressive symptoms and frailty phenotypes. The findings suggest that depressive symptoms and frailty phenotypes among community-dwelling older Filipinos are two plausible explanations for the experience of sleep deficiency when suffering from food insecurity. These associations were beyond the relationships of sociodemographic characteristics, health, and health behaviours with sleep deficiency.

Similar to previous studies [15,16], the results indicate that depressive symptoms significantly mediate the relationship between food insecurity and sleep deficiency among older adults. In terms of food intake, individuals who experience food insecurity may have poorer diets, which can lead to imbalances in nutrients, such as magnesium, tryptophan, and melatonin, all of which are important for promoting healthy sleep [50]. Moreover, depression has been shown to increase nighttime wakefulness, disrupt sleep patterns, and decrease sleep efficiency [50]. This disruption may be exacerbated by the stress of food insecurity (*e.g.* concerns about food or financial inability to obtain food), eliciting the hypothalamic-pituitary-adrenal axis – a key role in regulating the body’s stress response and sleep-wake cycle [51], leading to a downward spiral where a higher number of depressive symptoms aggravating the risks of sleep deficiency.

The findings also contribute to the literature by demonstrating that frailty phenotypes potentially mediate the relationship between food insecurity and sleep deficiency among older adults. Research has shown that older adults who experience food insecurity are less physically active [52], walk slower [53], and have poor nutritional status [22,23] and weaker strength [54], which are relevant to all frailty phenotypes [31]. Nevertheless, food insecurity may not necessarily lead to weight loss. According to the food insecurity-obesity paradox [55], food insecurity might result in obesity because individuals consume more energy-dense food. Additional analysis in the current sample showed that food insecurity was significantly positively associated with underweight (no food insecurity 12% *vs.* food insecurity 14.0%) and pre-obesity (no food insecurity 25.0% *vs.* food insecurity 28.0%) but negatively associated with obesity (no food insecurity 9.0% *vs.* food insecurity 3.0%). These findings suggest that food insecurity may not necessarily lead to being underweight, but it could also result in pre-obesity or obesity. Because the relationship between food insecurity and weight changes may depend on many other factors, such as social (*e.g.* affordability of energy-dense food) [56] and physiological factors (*e.g.* the proportion of body fat affects the relationship between BMI and frailty) [55,56], exploring these relationships is beyond the scope of the current study. Hence, we recommend that future research understand the link between food insecurity and frailty phenotypes and how these lead to sleep problems. In the analysis, BMI in the mediation analysis was controlled, and the indirect effects of frailty phenotypes remained significant, suggesting that the mediating effects of frailty phenotypes were independent of BMI level.

The present study has important practical implications. Because of the negative consequences of food insecurity (*i.e.* having a higher number of depressive symptoms and frailty phenotypes) and its negative relationship with sleep problems, which are likely to yield a host of adverse health outcomes [57–60] and increase economic burdens [8], food insecurity among older adults should be a top priority in social protection programs (*e.g.* community-based health insurance and conditional cash transfer) [61]. Previous studies have shown that interventions such as a poverty-targeted social protection programme or a home delivery produce prescription programme [62] can effectively alleviate household food insecurity by improving food security [61]. In addition to these social interventions, screenings for mental health and frailty phenotypes among older adults who are food insecure or at risk of food insecurity should be implemented to prevent the development of sleep deficiencies. Moreover, education programmes about food insecurity may raise awareness of the impacts of food insecurity on mental health, frailty, and sleep among older adults, caregivers, and family members.

The present work is the first attempt to examine the mediating role of depressive symptoms and frailty phenotypes simultaneously in the relationship between food insecurity and sleep deficiency in older adults, providing empirical support for studying the relationship between food insecurity and sleep deficiency in this population. The strength of this study is that it has a large sample size from a nationally representative sample of community-dwelling older Filipinos. Nonetheless, this study is subject to several limitations. The first is due to the cross-sectional nature of the study design. Therefore, conclusions about causality should be interpreted with caution. This limitation is particularly relevant when considering the potential bidirectional nature of the relationships among food insecurity, depressive symptoms, frailty phenotypes, and sleep deficiency [35,63]. Future studies should replicate the current study with a longitudinal design. Second, food insecurity was measured using a single item, although a single-item assessment strategy was shown to detect household food insecurity with high specificity and sensitivity [64,65]. The drawback of the single-item strategy is that it can only provide a global assessment of food insecurity that might not reflect the multidimensional nature of food insecurity. Hence, future research is recommended to use assessments, such as the Food Insecurity Experience Scale [66], to evaluate food insecurity. In addition, the single item focuses on food insecurity at the household level. Although individuals living in a household that suffers from food insecurity are vulnerable to food insecurity, food allocation, and access to additional food outside of the household may play a role at the individual level [67]. Hence, future research is recommended to investigate the differential impacts of individual- and household-level food insecurity on sleep deficiency among older adults. Another relevant issue was that the LSAHP did not have information on dietary intake. Hence, the current study could not further investigate whether dietary intake affects the relationship between food insecurity and sleep deficiency. Third, self-report items were used from archival data to conceptualise frailty and sleep deficiency. Future studies are recommended to use validated tools to assess frailty [39] and sleep [68]. Fourth, a predetermined list of chronic diseases was utilised. Although these diseases are common among older adults, individuals may have other chronic conditions that are not on the list. Furthermore, older adults may have taken drugs to manage their chronic diseases. However, no specific information was available on the medication that older adults were taking. Fifth, although the LSAHP collected information on sleep medication/treatment (‛In the past two weeks, have you taken any medications or used other treatments to help you sleep?’ (yes/no)), this variable was not included as a covariate because only 0.9% of the respondents reported using sleep medication/treatment. Adding this variable did not change the mediation effects (Table S4 in the **Online Supplementary Document**). There is a possibility that older adults may be taking sleep medications, but they do not know that they are taking them. For instance, antidepressants are prescribed for depression, but also for sleep problems. Therefore, future research should include a medication review to better understand the role of sleep medication in the relationship between food insecurity and sleep deficiency. Lastly, the findings can only be generalised to community-dwelling older Filipinos.

## CONCLUSIONS

In this study, we suggest an association between food insecurity and sleep deficiency, with frailty and depressive symptoms potentially mediating the relationship among older Filipinos living in the community. Therefore, targeted interventions to prevent frailty and depression could help improve sleep health in individuals experiencing food insecurity.

## Additional material


Online Supplementary Document
