# Supplementary material for: Food insecurity and sleep deficiency among older Filipinos: the mediating roles of depressive symptoms and frailty phenotypes
Source: J Glob Health. 2025 Aug 29;15:04235. doi: 10.7189/jogh.15.04235 (PMC12396318; doi:10.7189/jogh.15.04235)
Supplement: Online Supplementary Document [file jogh-15-04235-s001.pdf]

Supplement to: Domingo DGC, Chen TY, Cruz GT, Mgabhi PS, Lim KL, Mandiwa C, Saito Y. Food insecurity and sleep deficiency among older Filipinos: the mediating roles of depressive symptoms and frailty phenotypes. J Glob Health. 2025;15:04235.

Table S1. Operationalization of frailty

	Survey item	Frail	Not frail
Weight lost	Frequency of having poor appetite in the past 7 days	Often	Rarely/not at all<br>Sometimes
Exhaustion	Frequency of feeling everything is an effort<br>Could not get going in the past 7 days	Often	Rarely/not at all<br>Sometimes
Low physical activity	Frequency of engaging in physical exercises (e.g., walk, calisthenics, ballroom dancing, gardening)	Never<br>Few times a year<br>About Once a month	Every day<br>Several times per week<br>About once a week
Slowness	Perceived difficulty of walking 200-300 meters	Difficulty	No difficulty
Weakness	Perceived difficulty in lifting or carrying 5 kg of grocery	Difficulty	No difficulty

Table S2. Correlational analysis among variables

	1	2	3	4	5	6	7	8	9	10	11	12	13	14	15	16
1. Sleep deficiency (yes)	1															
2. Food insecure (yes)	.13**	1														
3. Depressive symptoms (0-14)	.20**	.14**	1													
4. Frailty phenotypes (0-5)	.09**	.15**	.18**	1												
5. Age (years)	-.01	-.09**	.08**	.18**	1											
6. Female (yes)	.09**	.04*	.11**	.18**	.03	1										
7. Education (1-3)	-.01	-.13**	-.20**	-.20**	-.17**	-.04*	1									
8. Live alone (yes)	.06**	.04**	.12**	.01	.13**	.02	-.12**	1								
9. Live in urban area (yes)	.02	-.05**	-.18**	.03*	.01	.07**	.18**	-.04*	1							
10. Wealth index (1-5)	-.07**	-.31**	-.29**	-.04**	-.02	.01	.39**	-.25**	.40**	1						
11. Pain (yes)	.14**	.13**	.31**	.23**	.06**	.02	-.10**	.02	-.10**	-.15**	1					
12. Chronic conditions (0-10)	.12**	.04**	.09**	.27**	.08**	.14**	.09**	-.11**	.12**	.20**	.19**	1				
13. BMI (0-3)	-.07**	-.03*	-.04**	.11**	-.08**	.19**	-.00	-.04**	.02	.09**	-.01	.19**	1			
14. Nap (yes)	-.08**	.05**	-.13**	-.01	.09**	-.08**	.05**	-.11**	-.00	.17**	-.06**	.09**	.02	1		
15. Smoking (yes)	-.03*	.10**	-.06**	-.07**	-.03	-.55**	-.06**	.07**	-.04*	-.11**	-.04**	-.13**	-.12**	.08**	1	
16. Drinking (yes)	-.05**	.11**	.04*	-.11**	-.04*	-.57**	-.05**	.05**	-.21**	-.17**	.02	-.18**	-.13**	.02	.45**	1

Note.

Table S3. Regression models for each pathways

	Frailty Phenotypes (M1)		Depressive Symptoms (M2)		Sleep Deficiency (Y)	
	B	*SE*	*B*	*SE*	*B*	*SE*
Food insecure (yes)	0.20***	0.05	0.76***	0.12	0.32	0.16
Frailty Phenotypes (0-5)	-	-	-	-	0.15**	0.05
Depressive Symptoms (0-14)	-	-	-	-	0.12***	0.02
Age	0.03***	0.002	0.001	0.005	-0.01	0.01
Female (yes)	0.31***	0.05	0.41**	0.10	-0.01	0.12
Education						
High school (ref: Elementary or lower)	-0.13**	0.04	-0.28**	0.09	0.01	0.11
College or above (ref: Elementary or lower)	-0.19**	0.06	-0.29*	0.14	-0.01	0.16
Living alone (yes)	-0.01	0.05	0.56***	0.11	0.13	0.14
Live in urban area (yes)	0.05	0.04	-0.15	0.08	-0.11	0.10
Wealth index (1-5; higher is wealthier)	-0.02	0.02	-0.22***	0.03	-0.03	0.04
Pain						
Mild pain (ref: no pain)	0.27***	0.05	0.53***	0.12	0.51**	0.16
Moderate pain (ref: no pain)	0.49***	0.05	1.21***	0.10	0.44**	0.13
Severe pain (ref: no pain)	0.66***	0.11	1.33***	0.23	1.28**	0.47
Chronic conditions (0-11)	0.21***	0.01	0.13**	0.03	0.17**	0.04
Nap (yes)	-0.07	0.04	-0.24**	0.09	-0.17	0.12
BMI						
Underweight (ref: Normal)	0.12*	0.05	0.31**	0.11	0.11	0.13
Pre-obese (ref: Normal)	0.06	0.04	-0.24**	0.09	-0.01	0.11
Obesity (ref: Normal)	0.15*	0.07	-0.17	0.15	0.30	0.20
Smoking						
Ever smoked (ref: never smoke)	0.06	0.05	0.23*	0.10	0.13	0.13
Current smoker (ref: never smoke)	0.05	0.06	0.34**	0.13	-0.14	0.15
Drinking						
Ever drank (ref: never drink)	0.14**	0.05	0.03	0.11	0.12	0.13
Current drinker (ref: never drink)	-0.05	0.05	-0.14	0.10	-0.002	0.12

**p<0.05, **p<0.01, ***p<0.001*

Table S4. Mediation analysis adding sleep medication

Mediator	Effect of X on M (β)	SE	Effect of M on Y (β)	SE	Bootstrap Estimate (β)	SE	95% CI Lower	95% CI Upper
Depressive symptoms	0.76***	0.12	0.12***	0.02	0.09	0.02	0.05	0.15
Frailty phenotypes	0.21**	0.05	0.15***	0.05	0.03	0.01	0.01	0.06
Total indirect effects	-	-	-	-	0.12	0.03	0.08	0.18
Direct effect of food insecurity on sleep deficiency	-	-	-	-	0.32	0.16	-0.002	0.64

Note. The model adjusted for age, sex, education, living arrangement, living in urban area, wealth status, pain, chronic conditions, body mass index, nap habit, sleep medication or treatment (yes/no), smoking, and drinking.
**p < .01; ***p <.001
